# Loss of long‐term benefit from VIM‐DBS in essential tremor: A secondary analysis of repeated measurements

**DOI:** 10.1111/cns.13770

**Published:** 2021-12-05

**Authors:** Yutong Bai, Zixiao Yin, Yu Diao, Tianqi Hu, Anchao Yang, Fangang Meng, Jianguo Zhang

**Affiliations:** ^1^ Department of Neurosurgery Beijing Tiantan Hospital Capital Medical University Beijing China; ^2^ Beijing Key Laboratory of Neurostimulation Beijing China

**Keywords:** deep brain stimulation, essential tremor, long‐term effects, meta‐analysis, predictive factors, ventral intermediate nucleus

## Abstract

**Aims:**

Deep brain stimulation (DBS) in the ventral intermediate nucleus (Vim‐DBS) is the preferred surgical therapy for essential tremor (ET). Tolerance and disease progression are considered to be the two main reasons underlying the loss of long‐term efficacy of Vim‐DBS. This study aimed to explore whether Vim‐DBS shows long‐term loss of efficacy and to evaluate the reasons for this diminished efficacy from different aspects.

**Methods:**

In a repeated‐measures meta‐analysis of 533 patients from 18 studies, Vim‐DBS efficacy was evaluated at ≤6 months, 7–12 months, 1–3 years, and ≥4 years. The primary outcomes were the score changes in different components of the Fahn‐Tolosa‐Marin Tremor Rating Scale (TRS; total score, motor score, hand‐function score, and activities of daily living [ADL] score). Secondary outcomes were the long‐term predictive factors.

**Results:**

The TRS total, motor, and ADL scores showed significant deterioration with disease progression (*p* = 0.002, *p* = 0.047, and *p* < 0.001, respectively), while the TRS total (*p* < 0.001), hand‐function (*p* = 0.036), and ADL (*p* = 0.004) scores indicated a significant long‐term reduction in DBS efficacy, although the motor subscore indicated no loss of efficacy. Hand‐function (*p* < 0.001) and ADL (*p* = 0.028) scores indicated DBS tolerance, while the TRS total and motor scores did not. Stimulation frequency and preoperative score were predictive factors for long‐term results.

**Conclusion:**

This study provides level 3a evidence that long‐term Vim‐DBS is effective in controlling motor symptoms without waning benefits. The efficacy reduction for hand function was caused by DBS tolerance, while that for ADL was caused by DBS tolerance and disease progression. More attention should be given to actual functional recovery rather than changes in motor scores in patients with ET.

## Graphical abstract

The firstly reported ventral intermediate nucleus has different effects on different aspects and provides high‐level evidence of the long‐term efficacy change of Vim‐DBS.

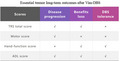

## 1 INTRODUCTION

Essential tremor (ET) is the most common type of pathologic tremor, with a prevalence of nearly 5% in elderly individuals.^1^, ^2^, ^3^ Pharmacotherapy is the primary treatment for most patients.^4^ However, it is only effective in 50% of patients.^5^, ^6^, ^7^ Surgical treatment is required for drug‐refractory patients.^8^, ^9^ The U.S. Food and Drug Administration (FDA) approved the use of ventral intermediate nucleus deep brain stimulation (Vim‐DBS) for the treatment of ET in 1997.^8^ Since then, DBS has been widely accepted for the treatment of ET and has shown promising short‐term outcomes. Studies have reported that approximately 60% to 80% reduction in tremor can be realized within 1 year after deep brain stimulation (DBS).^10^, ^11^ However, the reported long‐term effects have been a topic of debate. Sandoe et al.^12^ reported that anterior electrode placement of DBS leads to long‐term beneficial outcomes over 3 years, while Pahwa et al.^13^ reported that Vim‐DBS was associated with a 65% improvement rate after 5 years of follow‐up. However, Shih et al.^14^ found that the treatment's benefits waned in approximately two‐thirds of patients after more than 5 years. Similarly, Lu et al.^15^ reviewed the literature and reported that the efficacy of Vim‐DBS diminished over the long term. Thus, they speculated that the long‐term efficacy of Vim‐DBS was unreliable.

The reason for the loss of efficacy of DBS has attracted much research attention, with the current debate being centered on two reasons. The first of these is DBS tolerance, in which the brain shows a loss of response to Vim‐DBS with the stimulation on (stim‐on).^16^ The mechanism of DBS tolerance may involve attenuation of synchronous inhibition of cerebellar fiber tracts.^17^ The second reason is disease progression, which is defined by an increase in scores in the stimulation off (stim‐off) state. However, the improvement in the stim‐on state over the findings in the stim‐off remains the same as before. Favilla et al.^18^ conducted a prospective cohort study, pointing out that the “loss of benefit” is also due to disease progression and cannot be attributed to DBS tolerance alone. Whether the effects of Vim‐DBS on ET diminish over the long term is inconclusive, and if so, the reasons for this decrease remain to be explored. In this regard, research accounting for the efficacy reduction of Vim‐DBS in detail has remained limited, and a summary of the prognoses of long‐term outcomes is needed.

To address this gap in the literature, the present study aimed to evaluate the treatment efficacy and disease progression at different time points in ET and to compare the long‐term and short‐term efficacy at both stim‐off and stim‐on statuses. The predictive factors for the long‐term efficacy of Vim‐DBS were also identified.

## 2 METHODS

### 2.1 Literature review

This study followed the Preferred Reporting Items for Systematic Reviews and Meta‐Analyses (PRISMA) guidelines,^19^ and the study design was based on the PICOS strategy. We reviewed relevant studies in four databases (PubMed, Embase, World of Science, and the Cochrane Library). The search terms used were “essential tremor” and “deep brain stimulation” in the title, abstract, or keywords. For ET, we searched for the following terms: essential tremor OR idiopathic tremor OR senile tremor OR benign tremor OR ET. For DBS, we searched for the following terms: deep brain stimulation OR electrical stimulation therapy OR neuromodulation OR DBS. The time frame was from January 1, 1999, to August 31, 2019. Only studies published in English and those involving human participants were included. We also cross‐referenced some important articles by searching for articles citing and cited by them. Two authors (BYT and YZX) independently reviewed all the studies. We excluded irrelevant articles by scanning the abstracts and then checked the full text of relevant studies to further confirm if they should be included. For studies conducted in the same institution that covered the same group of patients, we only included the latest study with the largest sample size.

### 2.2 Inclusion and exclusion criteria

The inclusion criteria were as follows: (1) the study participants were patients diagnosed with ET according to the consensus statement of the Movement Disorder Society^20^; (2) the patients were treated with Vim‐DBS; (3) the studies used the Fahn‐Tolosa‐Marin Tremor Rating Scale (TRS) to evaluate disease severity; and (4) the studies reviewed both preoperative and postoperative clinical data.

The exclusion criteria were as follows: (1) the study participants were also diagnosed with other tremors; (2) the study participants received other surgical treatments prior to Vim‐DBS; (3) more than two leads or more than one target nucleus were implanted in the patients; (4) the scale assessment was conducted online; (5) the studies only reported subitem scores, such as right limb posture scores or head scores; and (6) necessary data (mean or SD) were not reported.

### 2.3 Quality assessment

We used the Meta‐analysis Of Observational Studies in Epidemiology (MOOSE) guideline^21^ to assess the bias of observational studies when assessing the quality of studies with respect to the following six different aspects: (1) clearly defined study population with more than five properly diagnosed patients; (2) clearly defined outcomes and outcome assessment, which included the TRS total score and the motor, hand‐function, and activities of daily living (ADL) subscores; (3) outcome parameters assessed independently, with the assessor and the assesses remaining anonymous; (4) a sufficient follow‐up period lasting at least for 6 months; (5) no significant selective loss during follow‐up, with a loss rate less than 15%; and (6) identification of important confounders or prognostic factors (reporting baseline features). The total score ranged from 0 (lowest quality) to 6 (highest quality). Research scores of more than four were considered to indicate high quality. Details were in Table S2.

### 2.4 Data extraction

We extracted the following variables: study type (prospective or retrospective study), study institution, age at surgery, unilateral or bilateral DBS, medications, sex, duration, number of patients, preoperative TRS scores (TRS total score and motor, hand‐function, and ADL subscores), follow‐up time points, the four TRS scores at different postoperative time points, and programming parameters at the last follow‐up. The TRS scores were collected under two conditions: with stimulation (stim‐on) and without stimulation (stim‐off).^22^ We divided the follow‐up time points into four groups: 6 months, 7–12 months, 1–3 years, and >4 years.^23^ For each period, mean and SD values of the scores were extracted. For studies with no SD reported, we extracted the *p* value, standard error (SE), and the 95% confidence interval to estimate the SD.^24^ Two authors (BYT and YZX) extracted the data independently, and consensus was reached through discussion when disagreements occurred. If no consensus could be reached through discussion, the final decision was made by the corresponding author (ZJG).

### 2.5 Analysis process

This study was a meta‐analysis of single‐arm repeated measurements. We used the all‐time‐points meta‐analysis (ATM) and the change‐in‐time meta‐analysis (CTM) methods to calculate the differences between different time points.^25^ ATM is used to pool the data from all time points and compare it with the baseline. The advantage of ATM is that it compares the scores over several time points with the preoperative scores. In this study, we obtained data for four postoperative time points, and we used ATM to compare the corresponding scores with the baseline. CTM focuses on the changes between the estimates at successive time points. CTM can be performed in two ways: the differences between successive time points are calculated and combined,^26^ or the difference from baseline to each time‐point is calculated.^27^ Here, we used the second CTM method to compare the changes in differences between the two time points and the baseline (6 months and 4 years). Specifically, we first calculated the mean difference in TRS scores (TRS total scores, motor scores, hand‐function scores, and ADL scores) between different time points in different conditions (stim‐on/stim‐off) in comparison with the baseline. Then, we pooled the data for each time point (baseline, 6 months, 7–12 months, 1–3 years, and >4 years). Second, we used the TRS scores in the stim‐on condition to calculate the improvement rate in comparison with the baseline at different follow‐up time points. The TRS scores in the stim‐off condition were used to calculate the rate of disease progression, where positive values indicated disease deterioration and negative values indicated continued improvement. Then, we compared the improvement rate and disease progression rate at different time points with the baseline by using the ATM method. More importantly, we compared the long‐term outcomes (≥4 years) with short‐term outcomes (≤12 months) in the stim‐on condition to reveal the stability of DBS in ET by using the CTM method. Finally, we performed a meta‐regression to show which factors affected DBS improvement in the long term (4 years).

### 2.6 Statistical analysis

This study was registered in PROSPERO (CRD42020151511). All statistical analyses were performed using Comprehensive Meta‐Analysis Version 3.3 (Biostat). Data displayed only on graphs were extracted by the Web Plot Digitizer (https://automeris.io/WebPlotDigitizer/). To analyze standardized mean differences (SMDs) between FTM‐TRS scores at different time points, a corrected effect size (Hedges' g) was calculated for each study, wherein the pooled weighted standard deviations were employed to correct for the small sample size. Heterogeneity was assessed using the standard Cochrane Q and *I* ^2^ statistics. Because this study involved single‐arm analysis, we employed random‐effects models. Meta‐regression analysis was performed using the maximum likelihood method. Finally, publication bias was assessed using Egger's test. Differences were considered statistically significant at *p* < 0.05.

## 3 RESULTS

### 3.1 Literature review

**FIGURE 1:**
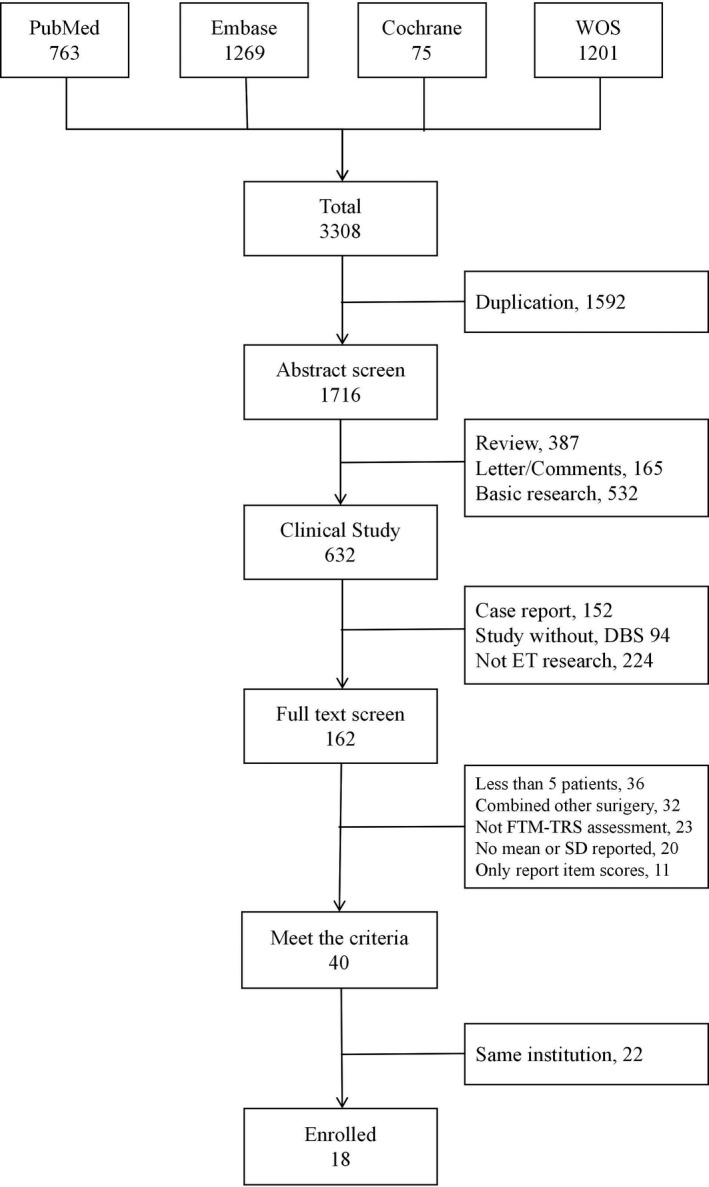
The flow of the literature search

The literature search yielded a total of 3308 articles from four main databases. Based on the inclusion criteria, 18 studies with 533 patients were included in our study. Figure 1 shows the flow diagram of the literature search. We reviewed all studies and summarized the baseline characteristics in Table 1. The average age of these patients was 67.7 years, and the mean ET duration was 27.5 years. Various methods of electrode positioning were employed in these studies, and the common steps were as follows: localization of the VIM by magnetic resonance imaging (MRI) fused with stereotactic framed head CT superimposed by an anatomic atlas, placement of the lead during the microelectrode recording (MER), and testing of the DBS effect intraoperatively. Approximately, 61% (11/18) of the studies carried out the entire process, while 28% (5/18) omitted the MER step and used MRI to localize the lead and tested the DBS effect by the intraoperative stimulation test (IST) subsequently. Only 11% (2/18) of the studies only reported MRI localization without describing any intraoperative testing. We extracted time points in all studies and sorted them into four groups (Table S1). Since the studies included different subscales of the TRS scores, we evaluated the publication bias and found no significant publication bias (Table S3).

**TABLE 1:** Baseline characteristics of the included studies

Study	Quality	Study type	Number	Age (year)	Disease duration (years)	MRI	MER	Macrostimulation
Paschen 2019^28^	6	Retrospective	20	67 ± 8	37 ± 17	√	√	√
Klein 2017^29^	4	Retrospective	26	67 ± 9	25 ± 17	√	×	×
Favilla 2012^30^	6	Retrospective	28	74 ± 11	37 ± 20	√	√	√
Heber 2013^31^	6	Prospective	9	66 ± 9	24 ± 16	√	×	√
Blomstedt 2007^32^	6	Retrospective	19	68 ± 7	23 ± 17	√	×	√
Rezaei 2017^33^	6	Retrospective	10	70 ± 19	32 ± 19	√	×	√
Rodríguez 2016^34^	5	Retrospective	14	61 ± 3	25 ± 11	√	√	√
Sydow 2003^35^	6	Retrospective	19	62 ± 11	38 ± 12	√	√	√
Fields 2003^36^	6	Prospective	40	72 ± 9	18 ± 13	√	×	√
Cury 2017^37^	6	Retrospective	38	64 ± 11	21 ± 13	√	√	√
Higuchi 2015^38^	5	Retrospective	44	66 ± 10	22 ± 14	√	√	√
Pahwa 2006^39^	5	Prospective	28	70 ± 5	NA	√	√	√
Putzke 2004^40^	6	Prospective	52	72 ± 8	25 ± 16	√	√	√
Rehncrona 2003^41^	5	Retrospective	19	66 ± 11	30 ± 14	√	×	√
Ondo 2001^42^	6	Prospective	13	72 ± 5	NA	√	NA	NA
Kumar 1999^43^	6	Retrospective	9	69 ± 10	26 ± 15	√	√	√
Vesper 2004^44^	5	Retrospective	18	NA	NA	√	√	√
Wharen 2017^45^	6	Prospective	127	65 ± 10	29 ± 17	√	√	√

Abbreviations: IST, intraoperative stimulation test; MER, microelectrode recording; MRI, magnetic resonance imaging.

### 3.2 Tremor Rating Scale scores at different time points

**FIGURE 2:**
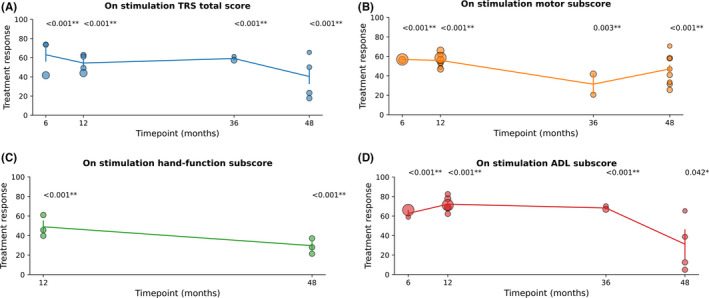
TRS scores at different time points on stimulation. (A) TRS total score, (B) motor subscore, (C) hand‐function subscore, and (D) ADL subscore. All scores at all time points were significantly different with preoperative scores. Individual results were presented by dots; the diameter of the dots reflect the sample size of the study. Only one study had a follow‐up period between 1 and 4 years (stim‐on) in hand‐function score. ADL, activities of daily living; TRS, Tremor Rating Scale

We analyzed the TRS total score and the motor, hand‐function, and ADL subscores in both stim‐on and stim‐off conditions at different time points. We first compared the follow‐up scores with the baseline in the stim‐on condition (Figure 2 and Table S4). The score changes were considered to reflect improvements during the follow‐up period. All subscores at all time points showed significant differences in comparison with the baseline. Long‐term efficacy was confirmed in the long‐term follow‐up, and the peak score showed a decreasing trend, although the decrease was statistically insignificant. In the hand‐function and ADL subscales, the average rate of improvement after 4 years was only about half of the maximum improvement rate.

**FIGURE 3:**
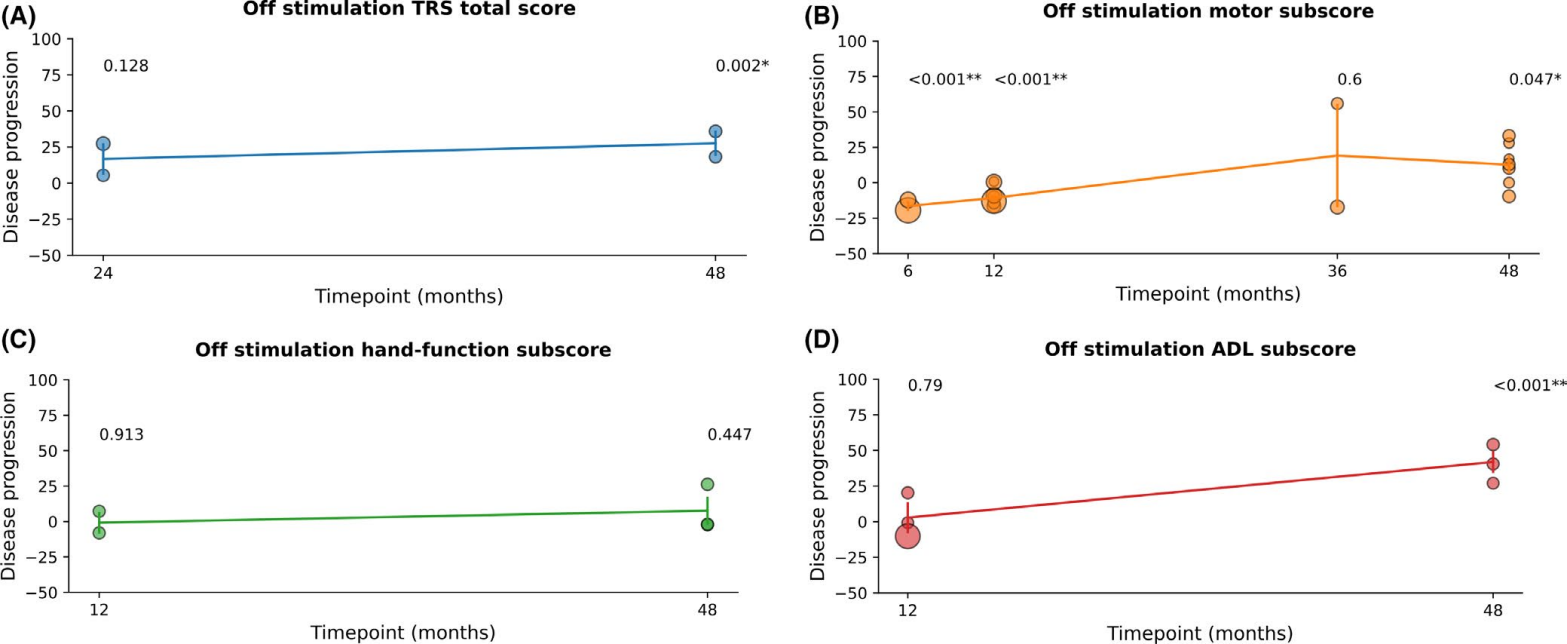
TRS scores at different time points of stimulation. (A) TRS total scores, (B) motor subscore, (C) hand‐function subscore, (D) ADL subscore. ETs progress significantly in both TRS total score and ADL subscore during the long‐term follow‐up. Motor subscore improved in the first year after DBS, while slightly progressed in the long‐term follow‐up. Hand‐function subscore had not seen significant progressing. Studies which reported stim‐off scores were fewer than stim‐on scores. Individual results were presented by dots, and the diameter of the dots reflect the sample size of the study. One study reported the 12‐month follow‐up TRS total scores, and another study reported the 24‐month results; we merge them together to calculate the disease progression within 2 years. Only one study had a follow‐up period between 1 and 4 years in hand‐function score, which was not included in the further analysis. No study reported ADL score in stim‐off between 1 and 4 years. ADL, activities of daily living; TRS, Tremor Rating Scale

The stim‐off results are shown in Figure 3 and Table S5. We evaluated the scores on the basis of the disease progression rate, and the findings for the different TRS scores varied widely. The TRS total score indicated progression after 24 months and significant worsening after 4 years. The motor subscore slightly decreased in the first 12 months but deteriorated significantly after 4 years. The hand‐function subscore showed no significant difference during the long‐term follow‐up. However, the changes in the ADL subscore were similar to those in the TRS total score, which remained stable for 12 months and deteriorated after 4 years.

### 3.3 Comparisons between short‐ and long‐term follow‐up findings

Of the 18 included studies, 9 reported both short‐term (<12 months) and long‐term (>4 years) results. We extracted these data and displayed the changes from the short to the long term (Table 2). We categorized these results as the loss of the effect of DBS. The motor subscore remained stable during the long‐term follow‐up, and it showed no significant difference during follow‐up (*p* = 0.183). However, the TRS total score and the other two subscores all indicated a reduction in the efficacy of DBS (*p* < 0.001, *p* = 0.036, and *p* = 0.004).

**TABLE 2:** Tremor Rating Scale (TRS) score comparison of the short‐term and long‐term efficacy of deep brain stimulation (DBS)

	TRS total score	(A) Motor subscore	(B) Hand‐function subscore	(C) ADL subscore
Studies	3	7	3	5
Point estimate	17.23	2.10	4.84	4.73
Standard error	2.11	1.57	2.31	1.63
Z‐value	8.17	1.33	2.09	2.91
*p* value	<0.001*	0.183	0.036*	0.004*

Note

This table compared the long‐term (>4 years) and short‐term (<12 months) outcomes to reflect the benefit loss of DBS in different aspects. TRS total score, hand‐function subscore, and ADL subscore all showed the benefit loss, while motor subscore kept stable during the long‐term follow‐up.

* Significant difference.

### 3.4 Comparisons between essential tremor disease progression and loss of deep brain stimulation benefits

In a subsequent analysis, we compared the loss of DBS benefits with ET disease progression (Table 3). DBS tolerance was considered to exist when the loss of benefits was significantly larger than ET disease progression. A significant difference was observed in the hand‐function (*p* < 0.001) and ADL (*p* = 0.028) subscores, but not in the TRS total score (*p* = 0.059) or the motor subscore (*p* = 0.075).

**TABLE 3:** The comparison between essential tremor (ET) disease progression and deep brain stimulation (DBS) benefit loss

	TRS total score	(A) Motor subscore	(B) Hand‐function subscore	(C) ADL subscore
Studies	2^a^	7	3	3^a^
Point estimate	4.91	−0.86	3.12	1.38
Standard error	2.60	0.48	0.84	0.63
Z‐value	1.89	−1.78	3.71	2.19
P value	0.059	0.075	<0.001*	0.028*

Note

This table shows the comparison between ET disease progression and DBS long‐term benefit loss. When benefit loss is significantly larger than disease progression, we considered the results as DBS tolerance. Hand‐function and ADL subscores suffer from DBS tolerance during the long‐term follow‐up.

^a^ Only included studies reported both stim‐off and stim‐on scores in long‐term results.

* Significant difference.

### 3.5 Meta‐regression for long‐term outcomes

**FIGURE 4:**
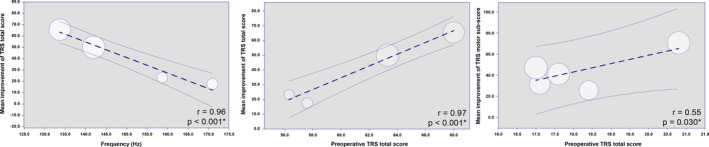
The results of meta‐regression. Frequency and preoperative Tremor Rating Scale (TRS) total scores are predictors of the improvement of TRS total score. Preoperative TRS total score is also the predictor of the improvement of TRS motor subscore

Baseline data were collected to determine the predictive factors influencing the long‐term outcomes (Figure 4). Due to the insufficient number of studies, we merely performed univariable meta‐regression. The predictive factors for the TRS total score were the frequency of stimulation (*r* = 0.96, *p* < 0.0001) and the preoperative score (*r* = 0.97, *p* < 0.0001). The preoperative score was also a predictive factor for the motor subscore. Frequency showed a negative correlation with the TRS total score, while the preoperative score showed a positive correlation with the TRS total score and motor subscore. No independent prognostic factors were observed for the hand‐function and ADL subscores.

## 4 DISCUSSION

The present investigation is, to our knowledge, the largest study to assess the long‐term efficacy of Vim‐DBS in the treatment of ET. A total of 533 cases from 18 studies were included in this investigation. We summarized the long‐term efficacy of Vim‐DBS in four parts (TRS total score, motor function, hand function, and ADL). We also discussed the reasons why Vim‐DBS lost its efficacy and explored the predictive factors for long‐term efficacy. The evidence obtained in this study suggests that Vim‐DBS is a promising treatment in terms of long‐term outcomes. The improvement rates of the four parts after a 4‐year follow‐up period were 40.4% (TRS total score), 47.1% (motor), 29.7% (hand function), and 31.1% (ADL). Efficacy loss was not observed in the motor score, indicating that motor capacity was well‐controlled and remained stable over the long term; in contrast, for hand function, the efficacy loss was due to DBS tolerance, and for ADL, the efficacy loss was due to disease progression (Table 4). The preoperative score and stimulation frequency were independent prognostic factors for long‐term clinical outcomes. Thus, we recommend that the efficacy of ET treatment should be confirmed from multiple perspectives instead of focusing solely on motor recovery. Improvement of both motor symptoms and actual functions will be a major challenge for future treatment.

**TABLE 4:** The long‐term outcome in each scores

Scores	Disease progression	Benefit loss	DBS tolerance
TRS total score	√	√	×
Motor score	√	×	×
Hand‐function score	×	√	√
ADL score	√	√	√

Note

This table summarized the results of our study.

### 4.1 Analysis of the Tremor Rating Scale total score and subscores

In our analysis, the efficacy of Vim‐DBS for ETs at all time points was significantly different from that at baseline. Previous studies have reported improvements in postoperative TRS scores in assessments of both short‐ and long‐term outcomes.^11^, ^13^, ^46^, ^47^ We verified that Vim‐DBS is a promising treatment for ETs. We further analyzed the disease progression of ET patients and concluded that the TRS total score worsened significantly. Two studies reflected a similar trend: 3.2%–5.3% ET progression per year.^48^, ^49^ Notably, the other subscores showed various changes. Motor scores were reduced by 10% to 16% within one year after surgery. The main cause could be the microlesion effect.^50^ Morishita et al.^51^ reported that the microlesion effect could control motor symptoms well in the first 6 months. Koller et al.^52^ reported a single case in which the microlesion effect was prolonged to 1 year. Our study found that this trend decreased from 6 months to 1 year after surgery, which confirmed our speculation. However, few studies have focused on hand function and ADL. We found that hand function showed no significant worsening during the long‐term follow‐up, and ADL progressed significantly. A study comparing 1 with 7 years of ADL scores postoperatively indicated that, except for eating, the efficacy of long‐term DBS on other aspects of ADL decreased.^53^

Whether Vim‐DBS loses its efficacy during the long‐term follow‐up is still under debate. According to the results of previous studies, the loss of DBS efficacy can be primarily attributed to three aspects: disease progression, DBS tolerance, and suboptimal lead position.^14^, ^18^, ^46^, ^54^ Favilla et al.^18^ reported that the TRS total score increased after 36 months of follow‐up, which means that disease progression is the main reason for benefit loss of DBS. Our study reached a similar result for the TRS total score. Interestingly, no significant difference was observed between the short‐term and long‐term outcomes in motor subscores. We inferred that Vim‐DBS maintained a stable effect on motor symptom control during the long‐term follow‐up. Some studies suggested that motor progress might be related to the rebound effect,^17^, ^55^, ^56^ and Steffen pointed out that evaluations performed 30 min after stimulation can largely eliminate the rebound effect.^57^ A total of 73% (8/11) of the enrolled studies that involved motor and TRS total scores mentioned a latency of 30 to 240 min between stim‐off and assessment, with a mean latency of 67.5 min. Therefore, we believe that the rebound effect did not significantly affect the results of this study. While benefit loss was also seen in hand function and ADL, this reduction in efficacy cannot be fully explained by disease progression. Therefore, we believe that these findings indicate the existence of DBS tolerance. The DBS tolerance associated with function has been less explored, and the results of this study suggest that the effects of DBS on patients may not be focused on motor symptoms but were more focused on causing functional impairment. Haubenberger et al.^58^ summarized that ET patients have a mild coordination dysfunction in limb movement, similar to ataxia, which may be responsible for the separation of motor and functional prognosis. Improvement of limb function may be a future goal in ET treatment.

We did not discuss the suboptimal location of leads because of the low homogeneity in the spatial coordinates provided by different articles. Sandoe et al.^12^ found that the anterior region of the Vim nucleus provided better clinical efficacy in ET patients and maintained efficacy for a long‐time period. Some experts have indicated that the location between leads does not affect benefit loss.^14^ Enlarging the volume of tissue activated by programming cannot solve this problem.^17^ In recent years, new targets such as PSA and DRTt have gradually matured in DBS surgery and have achieved good results.^53^, ^59^, ^60^, ^61^, ^62^, ^63^ However, Murata et al.^60^ reported that PSA may also cause a loss of DBS benefits. Anthofer et al.^64^ found that patients with a long distance from contact with DRTT fibers are more likely to experience DBS tolerance. Further research is needed to explain the benefit loss in terms of the locations of DBS leads.

### 4.2 Analysis of predictive factors

We then analyzed the predictive factors for the long‐term outcomes of Vim‐DBS. Preoperative score and frequency were predictive factors for the TRS total score. The preoperative score is a common factor in predicting the effect of clinical outcomes. Several articles have pointed out that patients with more severe symptoms show more reliable improvements.^8^, ^12^ A meta‐analysis showed that patients with low preoperative scores improved well, with a mean follow‐up period of only 20 months.^15^ They regressed the subitem scores together with the TRS total scores and obtained low homogeneity. Studies analyzing programming parameters are still limited. Our study pointed out that a high frequency could affect the effect of Vim‐DBS. Ramirez‐Zamora et al.^65^ reported that reduced frequency from 170–185 Hz to 130 Hz can improve the cerebellar axial symptoms of patients with ET, suggesting that a too high frequency causes side effects and reduced efficacy. Currently, research on frequency is mainly focused on high‐frequency stimulation, so no low‐frequency studies were included in our research. In Parkinson's disease research, several centers used 25–60 Hz for DBS treatment. Xie et al.^66^ considered that low‐frequency stimulation might not be able to control tremor or high‐frequency stimulation. Based on the results of our study, we suggest choosing an appropriate high frequency to control tremor and altering the possible side effects when the frequency is too high. Few studies have reported on the pulse width during the long‐term follow‐up. Therefore, this study did not analyze the relationship between pulse width and long‐term efficacy. In recent years, some studies have used short pulse width (40–60 µs) to treat patients with ET and have achieved certain results that might apply to the clinic in small sample sizes^67^, ^68^; further verification of this approach is required in future studies.

### 4.3 Limitations

Our study had several limitations. First, most of the included studies were observational studies aimed at assessing the results. Therefore, it was difficult to establish a prospective study for a period longer than 4 years. However, using the MOOSE method, we excluded studies with MOOSE scores lower than 4, thereby including only high‐quality studies. Moreover, no publication bias was observed in our study, which reinforced the credibility of the findings. Second, we did not assess all subitem scores of the TRS because of the insufficient sample size. Third, we did not evaluate the position of leads, since studies published in different years used different references, and no studies have reported long‐term follow‐up data for the movement of leads. The lead location in the Vim nucleus that shows the best clinical efficacy is still a topic of debate and worthy of further exploration.

## 5 CONCLUSION

This study provides level 3a evidence of the long‐term efficacy of Vim‐DBS. Over the long‐term follow‐up period, the effectiveness of Vim‐DBS differed for various aspects of recovery. Vim‐DBS was shown to be effective with no waning benefits in controlling motor symptoms. However, DBS tolerance led to an efficacy loss for hand function, and disease progression and tolerance were associated with the loss of efficacy for ADL. These findings indicate the need for greater attention to actual functional recovery rather than changes in motor scores in patients with ET.

## CONFLICT OF INTERESTS

The authors report no conflict of interest concerning the materials or methods used in this study or the findings specified in this paper.

## Supporting information

Table S1‐S5

## Data Availability

The data used to conduct statistics in this study are available from the corresponding author upon reasonable request.
