# Supplementary material for: Loss of long‐term benefit from VIM‐DBS in essential tremor: A secondary analysis of repeated measurements
Source: CNS Neurosci Ther. 2021 Dec 5;28(2):279–88. doi: 10.1111/cns.13770 (PMC8739044; doi:10.1111/cns.13770)
Supplement: Supplementary file 1 — Table S1‐S5 [file CNS-28-279-s001.docx]

| Study | Time points | | | | | | | | | | | | | | | |
| --- | --- | --- | --- | --- | --- | --- | --- | --- | --- | --- | --- | --- | --- | --- | --- | --- |
|  | ≤ 6 months | | | | 6-12 months | | | | 12-36 months (1-3 years) | | | | ≥ 48months (4 years) | | | |
|  | Total | A | B | C | Total | A | B | C | Total | A | B | C | Total | A | B | C |
| Deuschl 2019 |  |  |  |  | √ | √ | √ | √ |  |  |  |  | √ | √ | √ | √ |
| Kupsch 2017 |  |  |  |  |  |  |  |  | √ |  |  |  |  |  |  |  |
| Okun 2012 |  |  |  |  |  | √ |  | √ |  | √ |  | √ |  |  |  |  |
| Heber 2013 |  |  |  |  |  | √ |  |  |  |  |  |  |  | √ |  |  |
| Patric 2007 |  |  |  |  | √ | √ | √ | √ |  |  |  |  | √ | √ | √ | √ |
| Haddad 2017 |  |  |  |  |  |  |  |  |  |  |  |  | √ | √ |  | √ |
| Francisco 2016 | √ |  |  |  |  |  |  |  |  |  |  |  | √ |  |  |  |
| Sydow 2003 |  |  |  |  |  | √ | √ | √ |  |  |  |  |  | √ | √ | √ |
| Tröster 2003 |  | √ |  |  |  | √ |  |  |  |  |  |  |  |  |  |  |
| Moro 2017 |  |  |  |  |  | √ |  |  |  |  |  |  |  | √ |  |  |
| Higuchi 2015 | √ |  |  |  | √ |  |  |  |  |  |  |  |  |  |  |  |
| Pahwa 2006 |  |  |  |  |  |  |  |  |  |  |  |  |  | √ |  |  |
| Putzke 2004 |  |  |  |  |  |  |  | √ |  |  |  | √ |  |  |  |  |
| Rehncrona 2003 |  |  |  |  |  |  |  |  |  | √ |  |  |  | √ |  |  |
| Ondo 2001 |  |  |  | √ |  |  |  |  |  |  |  |  |  |  |  |  |
| Kumar 1999 |  |  |  |  |  |  |  |  | √ |  | √ |  |  |  |  |  |
| Vesper 2004 | √ |  |  |  | √ |  |  |  |  |  |  |  |  |  |  |  |
| Wharen 2017 |  | √ |  | √ |  | √ |  | √ |  |  |  |  |  |  |  |  |
| Sum up | 3 | 2 | 0 | 2 | 4 | 8 | 3 | 6 | 2 | 2 | 1 | 2 | 4 | 8 | 3 | 4 |

Supplemental Table 1：The different time points contained in each studies

Supplemental Table 2: Assessment of risk of bias of included studies

| Study | Total score | Aspects | | | | | |
| --- | --- | --- | --- | --- | --- | --- | --- |
|  |  | Study population | Outcome | Independently assessment | Sufficient follow-up | Loss of follow-up | Confounders or prognostic factors |
| Deuschl 2019 | 6 | ★ | ★ | ★ | ★ | ★ | ★ |
| Kupsch 2017 | 4 | ★ | ★ |  | ★ | ★ |  |
| Okun 2012 | 6 | ★ | ★ | ★ | ★ | ★ | ★ |
| Heber 2013 | 6 | ★ | ★ | ★ | ★ | ★ | ★ |
| Patric 2007 | 6 | ★ | ★ | ★ | ★ | ★ | ★ |
| Haddad 2017 | 6 | ★ | ★ | ★ | ★ | ★ | ★ |
| Francisco 2016 | 5 | ★ | ★ |  | ★ | ★ | ★ |
| Sydow 2003 | 6 | ★ | ★ | ★ | ★ | ★ | ★ |
| Tröster 2003 | 6 | ★ | ★ | ★ | ★ | ★ | ★ |
| Moro 2017 | 6 | ★ | ★ | ★ | ★ | ★ | ★ |
| Higuchi 2015 | 5 | ★ | ★ |  | ★ | ★ | ★ |
| Pahwa 2006 | 5 | ★ | ★ |  | ★ | ★ | ★ |
| Putzke 2004 | 6 | ★ | ★ | ★ | ★ | ★ | ★ |
| Rehncrona 2003 | 5 | ★ | ★ | ★ | ★ |  | ★ |
| Ondo 2001 | 6 | ★ | ★ | ★ | ★ | ★ | ★ |
| Kumar 1999 | 6 | ★ | ★ | ★ | ★ | ★ | ★ |
| Vesper 2004 | 5 | ★ | ★ | ★ | ★ | ★ |  |
| Wharen 2017 | 6 | ★ | ★ | ★ | ★ | ★ | ★ |

Supplemental Table 3: Publication bias

| Rating scale | Studies | Z value | P value |
| --- | --- | --- | --- |
| TRS total score | 8 | 0.87 | 0.39 |
| motor subscore | 11 | 1.56 | 0.12 |
| hand-function subscore | 4 | 0.34 | 0.73 |
| ADL subscore | 8 | 1.61 | 0.11 |

Supplemental Table 4: TRS scores at different time points on stimulation

|  | TRS total score | | | | (A) motor subscore | | | | (B) hand-function subscore | | (C) ADL subscore | | | |
| --- | --- | --- | --- | --- | --- | --- | --- | --- | --- | --- | --- | --- | --- | --- |
| Time | ≤6m | 6-12m | 12-36m | >4y | ≤6m | 6-12m | 12-36m | >4y | ≤12m* | >4y | ≤6m | 6-12m | 12-36m | >4y |
| Number of Studies | 3 | 4 | 2 | 4 | 2 | 8 | 2 | 8 | 3 | 3 | 2 | 6 | 2 | 4 |
| Improving rate (%) | 63.08 | 54.46 | 59.17 | 40.39 | 56.78 | 55.88 | 31.44 | 47.08 | 48.96 | 29.66 | 62.66 | 72.18 | 68.27 | 31.14 |
| SE | 7.17 | 4.27 | 1.916 | 7.92 | 0.65 | 2.286 | 10.63 | 4.40 | 6.52 | 5.10 | 3.58 | 1.88 | 1.64 | 15.35 |
| Z-value | 8.80 | 12.75 | 30.88 | 5.10 | 86.86 | 24.44 | 2.96 | 10.69 | 7.505 | 5.81 | 17.49 | 38.49 | 41.62 | 2.03 |
| P value | <0.001 | <0.001 | <0.001 | <0.001 | <0.001 | <0.001 | 0.003 | <0.001 | <0.001 | <0.001 | <0.001 | <0.001 | <0.001 | 0.042 |

*Only one study had a follow-up period between 1 and 4 years (stim-on) in hand-function score, which was not included in the further analysis.

Supplemental Table 5: TRS scores at different time points off stimulation

|  | TRS total score | | (A) motor subscore | | | | (B) hand-function subscore | | (C) ADL subscore | |
| --- | --- | --- | --- | --- | --- | --- | --- | --- | --- | --- |
| Time | ≤24m* | >4y | ≤6m | 6-12m | 12-36m | >4y | ≤12m** | >4y | ≤12m*** | >4y |
| Number of Studies | 2 | 2 | 2 | 7 | 2 | 7 | 2 | 3 | 3 | 3 |
| Progressing rate (%) | 16.73 | 27.54 | -16.51 | -10.75 | 19.17 | 12.72 | -0.83 | 7.61 | 2.86 | 41.90 |
| SE | 10.98 | 8.81 | 3.60 | 2.30 | 36.55 | 6.40 | 7.64 | 9.99 | 10.96 | 7.69 |
| Z-value | 1.52 | 3.13 | -4.58 | -4.68 | 0.52 | 1.99 | -0.11 | 0.76 | 0.26 | 5.45 |
| P value | 0.128 | 0.002 | <0.001 | <0.001 | 0.6 | 0.047 | 0.913 | 0.447 | 0.79 | <0.001 |

Studies which reported stim-off scores were fewer than stim-on scores.

*One study reported 12 months follow-up TRS total scores and another study reported 24 months results, we merge them together to calculate the disease progression within 2 years.

**Only one study had a follow-up period between 1 and 4 years in hand-function score, which was not included in the further analysis.

***No study reported ADL score in stim-off between 1 and 4 years.
